# Dynamic Control of Band Alignment and Built‐In Potential in High Performance Self‐Powered InSe/SnS_2_ Van der Waals Photodetectors via Gas Molecular Physisorption

**DOI:** 10.1002/smsc.202500616

**Published:** 2026-03-06

**Authors:** Ze Cao, Mohamed Abid, Cormac Ó Coileáin, Fengjiang An, Ching‐Ray Chang, Yuh‐Renn Wu, Han‐Chun Wu

**Affiliations:** ^1^ School of Physics Beijing Institute of Technology Beijing P. R. China; ^2^ EduAI dynamics Astana Kazakhstan; ^3^ Department of Electrical Power Systems and Information Technology Institute of Physics University of the Bundeswehr Munich Neubiberg Germany; ^4^ State Key Laboratory of Explosion Science and Safety Beijing Institute of Technology Beijing P. R. China; ^5^ Department of Physics National Taiwan University Taipei Taiwan, ROC; ^6^ Quantum Information Center Chung Yuan Christian University Taoyuan Taiwan, ROC; ^7^ Graduate Institute of Photonics and Optoelectronics and Department of Electrical Engineering National Taiwan University Taipei Taiwan, ROC

**Keywords:** band alignment, built‐in potential, gas physisorption, InSe/SnS_2_, self‐powered photodetectors, van der waals heterojunction

## Abstract

Molecular physisorption provides a versatile strategy to dynamically tailor the optoelectronic properties of van der Waals (vdW) heterostructures, enabling extended carrier lifetimes, broadened spectral response, and erasable memory effects in self‐powered photodetectors. Here, we report how NO_2_ physisorption precisely modulates band alignment and built‐in potentials in self‐powered InSe/SnS_2_ heterojunction photodetectors. Using electrostatic gating, we identify three distinct regimes: (I) a robust p–n configuration (*V*_g_ ≤ –50 V), where adsorption induces a collective electron‐withdrawing effect, enabling efficient p‐i‐n‐like behavior with near‐ideal charge separation; (II) an intermediate p–n regime (–50 V < *V*_g_ < –30 V), where competing electron withdrawal and recombination effects allow dynamic tuning the electronic structure and optoelectronic properties, and (III) an illumination‐sensitive n–n^+^ mode (*V*_g_ ≥ –30 V), where NO_2_ molecules act as recombination centers, suppressing the built‐in potential. This dual control via gating and molecular adsorption provides unprecedented manipulation of charge separation and transport, opening avenues for next‐generation multifunctional optoelectronic devices.

## Graphical abstract

**Built‐in potential,** originating from band alignment, is the heart of the van der Waals (vdW) self‐powered heterojunction photodetectors. By leveraging NO_2_ physisorption, we dynamically tune the band alignment and built‐in potential in self‐powered InSe/SnS_2_ heterojunction photodetectors. This approach enables unprecedented control over charge separation and transport, paving the way for next‐generation multifunctional optoelectronic devices based on vdW heterostructures.

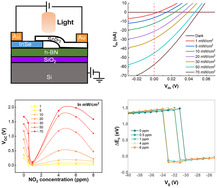

## 1 Introduction

Self‐powered photodetectors mark a transformative leap in optoelectronics by eliminating external power requirements while enabling ultra‐low energy consumption, compact integration, and enhanced portability [1]. Among the most promising materials for these devices are van der Waals (vdW) semiconductors [2, 3, 4, 5, 6], which exhibit exceptional light–matter interactions [7, 8], tunable bandgaps [9], and strong excitonic effects [10]. Critically, their weak interlayer vdW forces enable the precise assembly of heterojunctions with tailored interfacial properties—bypassing traditional lattice‐matching constraints [11, 12, 13, 14, 15]. Numerous self‐powered vdW photodetectors have been demonstrated [15, 16, 17], yet challenges persist, including interfacial defect‐induced recombination, narrow spectral responses, and scalability limitations. At the heart of self‐powered operation in these devices is the built‐in potential, originating from band alignment at heterojunctions. It is this field that ensures efficient separation of photogenerated carriers, enabling bias‐free operation with rapid charge extraction [18, 19, 20, 21]. This built‐in potential can be leveraged for peak performance using optimized vdW heterostructures [16, 22, 23, 24] or by material engineering, as demonstrated by recent advances in interface engineering [25], doping modulation [26], and integration with ferroelectrics [27] or piezoelectric [28, 29], thus offering viable solutions to the aforementioned challenges, and so making broadband photodetection with high responsivities and sub‐millisecond response times a real possibility.

Within the context of these developments, tin disulfide (SnS_2_) is particularly attractive as a low‐cost, nontoxic, and largely environmentally benign semiconductor, which combines high carrier mobility with strong light absorption [30], excellent photoresponse characteristics [30, 31, 32], and a layer‐independent bandgap [33]. Indium selenide (InSe), with its exceptional electrical [34, 35, 36], optical [34, 37, 38, 39], and spintronic [40, 41, 42] properties, is likewise a strong candidate for constructing high‐performance 2D p–n junctions with SnS_2_. Notably, SnS_2_/InSe heterostructures have been reported to exhibit type‐II band alignment, along with molecular physisorption‐induced erasable memory effects, highlighting the potential for multifunctional device architectures within a single device [43]. More recently, a bio‐inspired visual system was realized using Ta_2_NiSe_5_/SnS_2_ heterojunction, in which interfacial molecular physisorption extended carrier lifetimes and broadened the spectral response into the near‐infrared regime [44]. These advances bring to the fore a fundamental yet unresolved question: namely can the built‐in potential in InSe/SnS_2_ vdW heterostructures be effectively modulated through molecular physisorption? If so, then to what extent can such molecularly engineered interfaces enhance the efficiency and multifunctionality of self‐powered operation? Explaining this phenomenon is critical to determining the extent to which such molecularly engineered interfaces can enhance the efficiency and multifunctionality of self‐powered photodetectors as the interface is the device. Many frequently used gases (O_2_, H_2_S, NH_3_, CO_2_) typically demonstrate weak electron affinity, poor interaction stability, and low response efficiency—characteristics that hinder precise regulation and quantitative analysis—thus the response with respect to NO_2_ stands out, making it a superior candidate for practical experimental purposes [45]. Its strong electron acceptor properties [46] induce significant, yet reversible, charge transfer at 2D heterojunction interfaces while generating localized states near the bandgap center [47, 48, 49, 50]. Furthermore, as a prevalent environmental pollutant, the high‐sensitivity detection of NO_2_ has substantial practical importance [45]. These combined advantages make NO_2_ physisorption an obvious choice for modulating the band alignment and built‐in potentials in InSe/SnS_2_ photodetectors.

In this work, we report on the fabrication of self‐powered vdW InSe/SnS_2_ photodetectors and systematically uncover the critical role of molecular physisorption in modulating their optoelectronic response. Specifically, we demonstrate that NO_2_ physisorption can effectively tune both the band alignment and built‐in potential of InSe/SnS_2_ photodetectors. Three distinct operational regimes based on gate voltage are identified: In regime I (*V*_g_ ≤ −50 V), the junction operates in p–n mode, enabling efficient separation of photogenerated carriers. The built‐in potential is predominantly determined by the large conduction and valence band offsets and remains nearly constant (open circuit voltage *V*_OC_ ~ 2 V) regardless of light intensity. NO_2_ physisorption enhances the built‐in field. In regime II (−50 V < *V*_g_ < −30 V), the junction operates in p–n mode, and photogenerated carrier accumulation at the interface enhances the built‐in potential without NO_2_ physisorption. Upon exposure to NO_2_, both the band alignment and built‐in potential become highly tunable with gas concentration, offering dynamic control over device characteristics. In regime III (−30 V ≤ *V*_g_), the junction operates in n–n^+^ mode, where the built‐in potential increases with illumination intensity but is significantly weaker compared to the p–n regime. NO_2_ physisorption decreases the built‐in potential. This gate‐tunable and gas physisorption controlled feature presents a promising avenue for the development of high‐performance all‐in‐one device based on self‐powered heterojunction photodetector.

## 2 Results and Discussion

**FIGURE 1:**
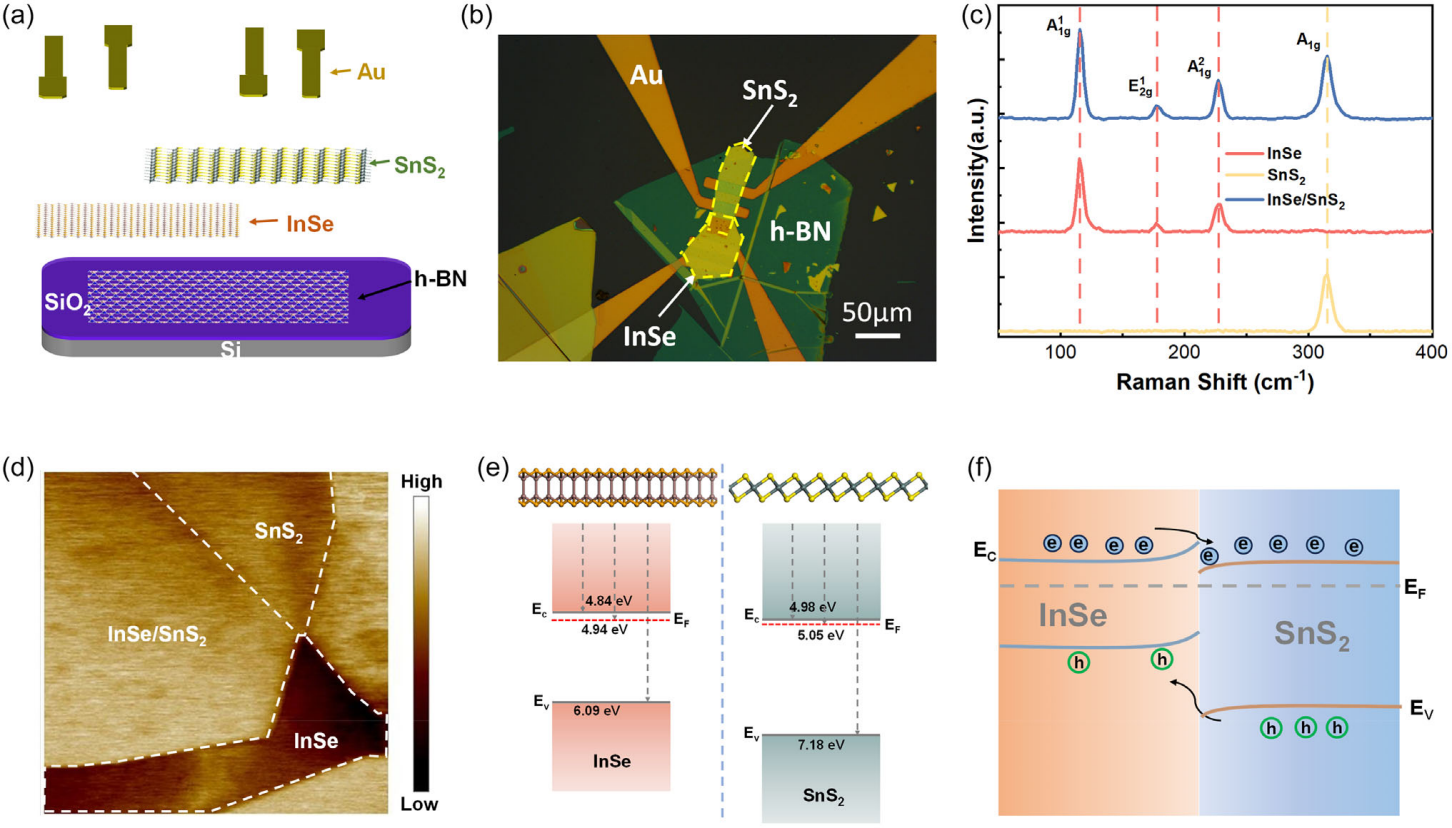
(a) Schematic diagram of device fabrication procedure. (b) Optical image of a typical fabricated InSe/SnS_2_ heterostructure photodetector. (c) Raman spectra of InS_2_, SnS_2_, and overlapping InSe/SnS_2_ areas under 532 nm laser excitation. (d) SKPM characterization of fabricated InSe/SnS_2_ vdW heterostructure. (e) Schematic diagram of the energy band of the InSe and SnS_2_ before contact. (f) Schematic diagram of the energy band and charge transfer of InSe/SnS_2_ heterostructure at *V*_g_ = 0 V.

Figure 1a,b illustrates the device fabrication procedure and an optical image of a typical InSe/SnS_2_ heterojunction. The device was fabricated using mechanical exfoliation, dry transfer, and UV lithography techniques. To suppress parasitic charge transfer from substrate dopants and improve electrostatic tunability and long‐term stability, a thin h‐BN film was used, transferred onto a Si substrate with a 300 nm SiO_2_ layer [51, 52]. For assembly, h‐BN, InSe, and SnS_2_ nanoflakes were mechanically exfoliated from their bulk single crystals, and the InSe/SnS_2_ heterojunction was built by vertically stacking, using a dry transfer method to preserve interfacial integrity. Details of the device fabrication can be found in the experimental section. Atomic force microscopy (AFM) was employed to characterize the thickness and morphology of the InSe and SnS_2_ layers, which revealed uniform surfaces with well‐defined edges (Figure S1). Raman spectra of the individual InSe, SnS_2_, and their overlapping portions were obtained under 532 nm excitation (Figure 1c). The characteristic peaks of the InSe spectrum appear at 115, 177, and 277 cm^−1^, corresponding to the $A_{1g}^{1}$, $E_{2g}^{1}$, and $A_{1g}^{2}$ vibration modes of β‐InSe, respectively [53, 54]. SnS_2_ exhibited a dominant peak at 315 cm^−1^ corresponding the $A_{1g}$ mode [55, 56, 57]. In the overlapping regions, all the signature Raman features of both materials were simultaneously observed, attesting to the structural integrity of the stacked layers and the preservation of their individual vibrational fingerprints. To probe the built‐in potential and charge transfer in the fabricated InSe/SnS_2_ heterojunction, scanning Kelvin probe microscopy (SKPM) was employed to map the local potential and extract the work functions of InSe, SnS_2_, and their overlapping regions (Figure 1d). The work function of SnS_2_ was found to exceed that of InSe, and spatial nonuniformities in the SnS_2_ work function—particularly near the heterojunction edge—suggested pronounced interfacial charge redistribution. Gaussian fitting of the potential distribution yielded work function values of 4.94 eV for InSe and 5.05 eV for SnS_2_ individually (Figures S2 and 1e). This observation was further confirmed with electrical measurements (Figure S3). Note, the work function was calibrated with respect to a standard Au electrode, with a work function set to 5.10 eV. Given that bulk InSe has a direct bandgap of 1.25 eV [36, 42], both SnS_2_ and InSe behave as n‐type semiconductors at zero gate voltage. Since the work functions of the individual InSe and SnS_2_ layers are 4.94 and 5.05 eV, respectively, electrons will transfer from the InSe layer to SnS_2_ layer while holes migrate in the opposite direction. Indeed, in the overlapping region, a work function of 5.04 eV was measured, indicating electrons really transfer from the InSe layer to the SnS_2_ layer (Figure 1f). This charge transfer is characteristic of a type‐II band alignment configuration with a built‐in potential pointing from InSe to SnS_2_. In addition, the conduction band offset $\left(\text{Δ}E_{C}\right)$ and the valence band offset $\left(\text{Δ}E_{V}\right)$ are 0.04 and 0.99 eV, respectively. These offsets also indicate that SnS_2_ provides a more energetically favorable conduction band for electrons while InSe offers greater stability for holes in the valence band. Consequently, upon contact, electrons preferentially transfer to SnS_2_ and holes migrate to InSe. This charge redistribution creates a built‐in electric field that drives carrier drift until the system reaches dynamic equilibrium.

**FIGURE 2:**
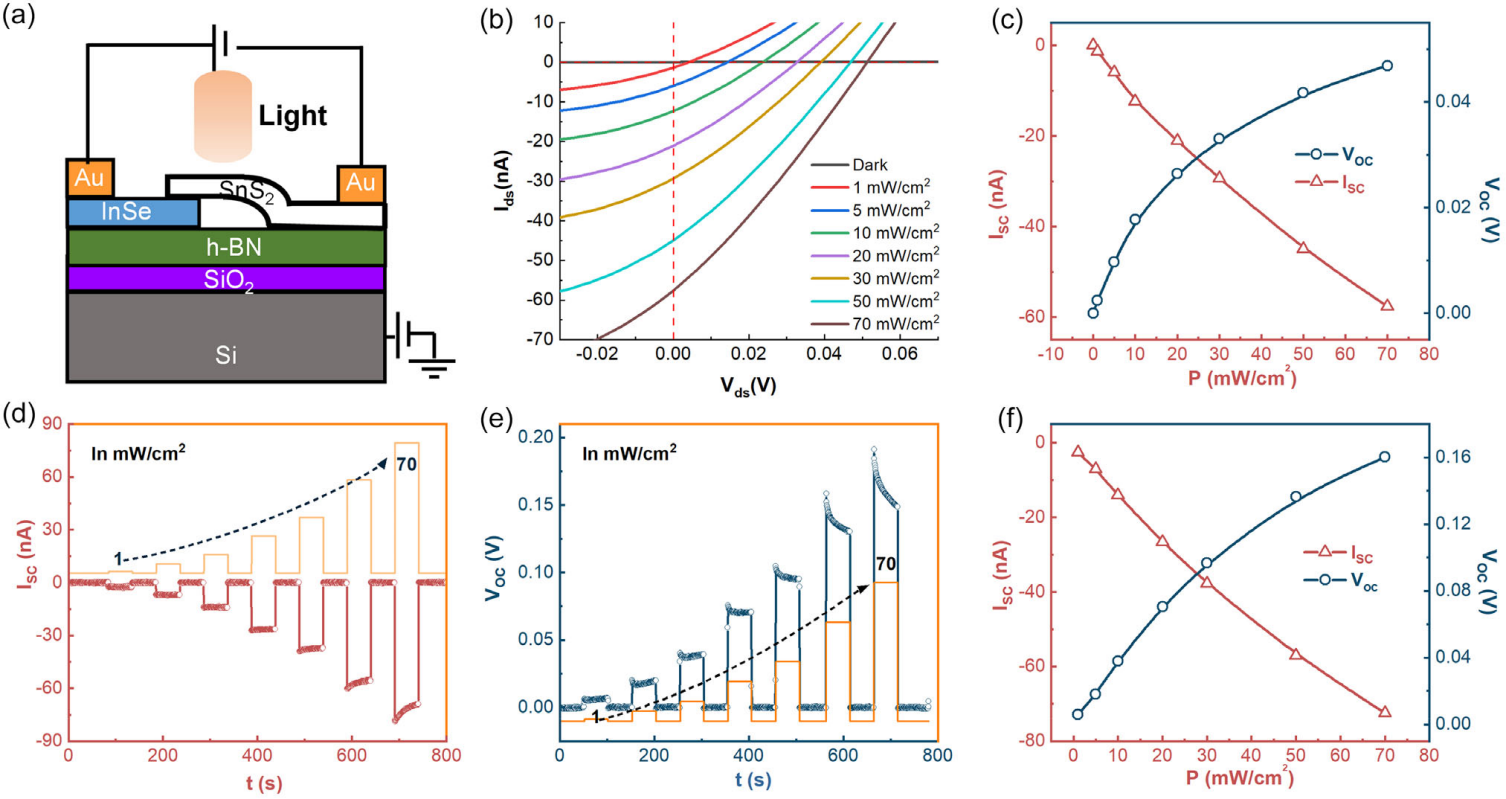
(a) Schematic of InSe/SnS_2_ vdW heterojunction device. (b) *I*_ds_–*V*_ds_ curves of the InSe/SnS_2_ vdW heterojunction device measured under dark conditions and 365 nm light illumination with a variety of incident power densities (P). (c) Summarized *I*_SC_ and *V*_OC_ extracted from (b). (d,e) Time‐resolved *I*_SC_ and *V*_OC_ under periodic illumination cycles measured at different incident power densities ranging from 1 to 70 mW cm^−2^. (f) Summarized *I*_SC_ and *V*_OC_ extracted from (d) and (e), respectively.

SKPM characterizations confirmed a type‐II band alignment in our InSe/SnS_2_ vdW heterojunction devices, enabling their operation as self‐powered photodetectors with a measurable short‐circuit current (*I*_SC_) and open‐circuit voltage (*V*_OC_) under light illumination. To verify this behavior, we conducted systematic photoresponse measurements. Figure 2a illustrates a schematic diagram of the InSe/SnS_2_ vdW heterojunction device, while Figure 2b plots *I*_ds_–*V*_ds_ curves measured in darkness and under 365 nm light illumination. As expected, no *V*_OC_ is observed in the dark case. Under 365 nm light illumination, the *I*_ds_ *–V*_ds_ curves shifts toward higher bias voltage, exhibiting distinct *I*_SC_ and *V*_OC_. This confirms that photoexcited electrons migrate to the SnS_2_ while holes move to the InSe. It further suggests that the built‐in potential is really directed from InSe to SnS_2_. Figure 2c summarized *I*_SC_ and *V*_OC_ as a function of incident power density. Both *I*_SC_ and *V*_OC_ increase with increasing incident power density and reach 58 nA and 0.047 V, respectively, under an incident power density of 70 mW·cm^−2^. These results confirm the device's capability to operate in a self‐powered mode under ambient illumination.

To further demonstrate self‐powered performance, time‐resolved *I*_SC_ and *V*_OC_ were measured under periodic illumination cycles at varying incident power densities as shown in Figure 2d,e. Figure 2f summarizes these time‐resolved *I*_SC_ and *V*_OC_ measurements, showing trends consistent with those in Figure 2c. Notably, at 70 mW·cm^−2^, *I*_SC_ and *V*_OC_ reach 72 nA and 0.16 V, respectively. This enhancement primarily stems from the high channel resistance and the differences in measurement system impedances: dynamic measurements employed a Keithley 2182A nanovoltmeter (ultra‐high input impedance), while output curves were acquired using Keithley 2400 sourcemeters (relatively lower impedance comparable with the channel resistance). During subsequent experiments, both *I*_SC_ and *V*_OC_ were measured with periodic illumination cycles. Moreover, *I*_SC_ follows a power‐law dependence on incident power density P: $I_{\mathrm{SC}}∼P^{\alpha }$, where α (0.82) reflects charge recombination suppression efficiency [58, 59, 60, 61, 62, 63] (Figure S4). This relatively high α value indicates efficient photo‐induced charge carrier separation in our InSe/SnS_2_ vdW heterojunction devices due to the type‐II band alignment [52, 63, 64]. However, the small *V*_OC_ measured at zero gate bias suggests that the device behaves like an n–n^+^ junction, a consequence of a weak built‐in field in the depletion region.

**FIGURE 3:**
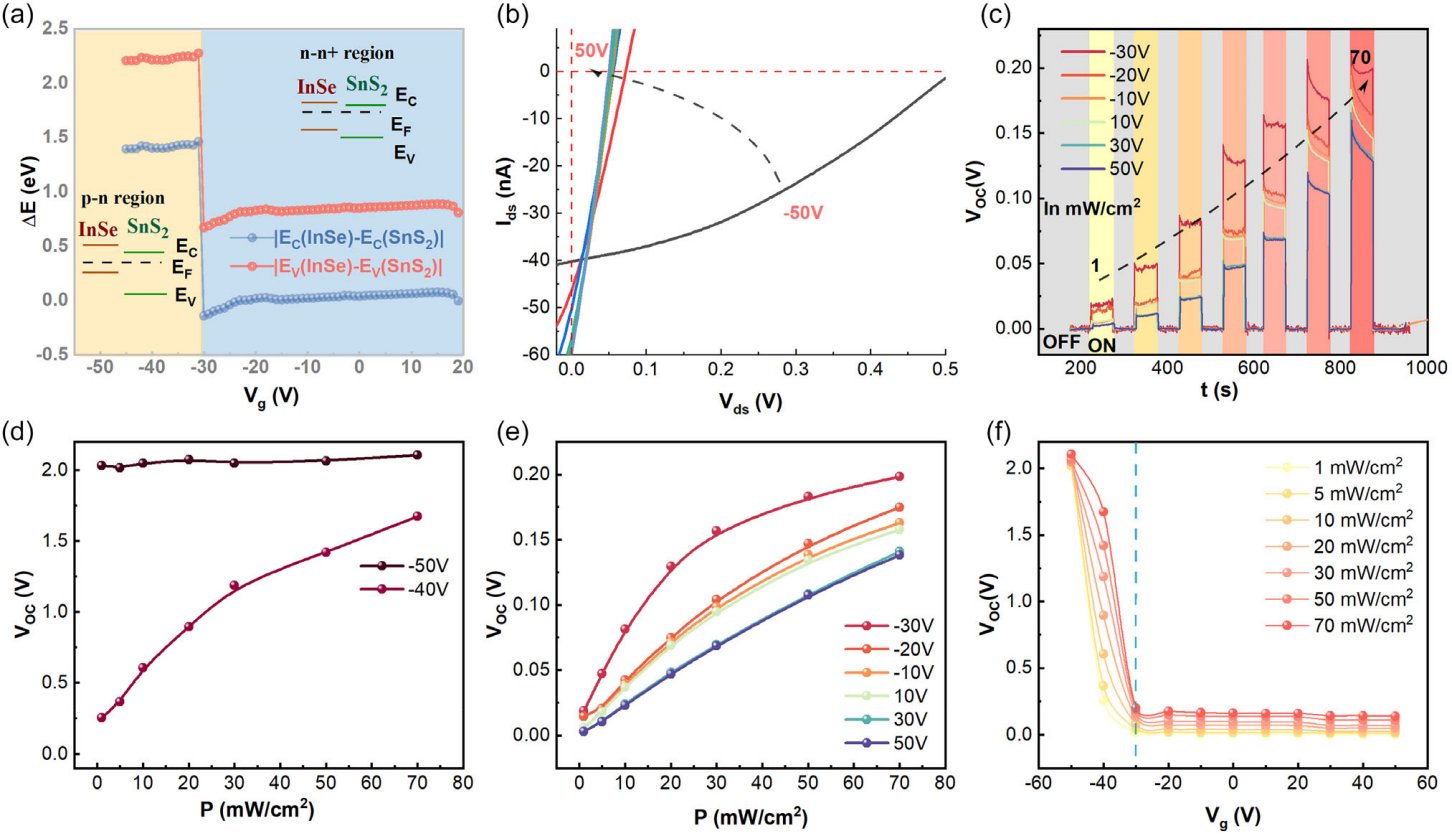
(a) The conduction band difference and valence band difference between InSe and SnS_2_ as a function of *V*_g_. (b) *I*_ds_ *–V*_ds_ measured at a variety of *V*_g_ under 365 nm light illumination with a constant incident power density of 70 mW cm^−2^. (c) Time‐resolved *V*_OC_ measured for a variety of *V*_g_ and with different incident power densities. (d,e) Summarized *V*_OC_ as a function of P extracted from (c). (f) Summarized *V*_OC_ as a function of *V*_g_ extracted from (c).

For a typical InSe/SnS_2_ vdW heterojunction device, the Fermi level of SnS_2_ remains relatively fixed [65], while that of InSe can be modulated across the entire band gap range by an external gate voltage (Figure S3). This allows extensive modulation of band alignments or the built‐in potential [43]. Specifically, a sufficiently negative gate bias can invert the charge carrier population in the ambipolar InSe layer, effectively transforming it into a p‐type region and forming a gate‐tunable p–n junction. Since the self‐powered performance of a heterojunction device depends crucially on the efficiency of photogenerated carrier separation and transport—both governed by the built‐in potential at the interface, we investigated the influence of an external electric field on the interfacial built‐in electrical filed. Figure 3a plots the conduction band difference (E_C_(InSe) – E_C_(SnS_2_)) and the valence band difference (E_V_(InSe) – E_V_(SnS_2_)) between InSe and SnS_2_ as a function of *V*_g_. Details can be found in the Supporting Information. Both band offsets exhibit pronounced shifts near *V*_g_ = –30 V, which we attributed to the ambipolar nature of the InSe (Figure S3). Consequently, there are two distinct operational regimes: p–n regime (*V*_g_ < *V*_Trans_) and n–n^+^ regime (*V*_g_ > *V*_Trans_), where *V*_Trans_ is the transition gate voltage. Notably, in the p–n regime, the conduction band difference is around 1.4 eV, and the valence band difference reaches 2.2 eV. These large band offsets imply a substantial built‐in potential, at least 1.4 V for electrons, across the junction. These large band potential differences also suggest a significantly high built‐in potential in the depletion region, which promotes efficient separation of photogenerated carriers in the p–n regime. To assess this effect, Figures 3b and S5a present *I*_ds_ *–V*_ds_ curves of a InSe/SnS_2_ vdW heterojunction device measured at a variety of *V*_g_ under 365 nm light illumination with a constant incident power density. The *V*_OC_ in the p–n regime is significantly greater than the values in n–n^+^ regime (Figure S5b), reaching 0.5 V at *V*_g_ = –50 V and exhibiting a pronounced decrease as *V*_g_ approaches the transition gate voltage *V*_Trans_. In contrast, in the n–n^+^ regime, *V*_OC_ is smaller than 0.05 V and exhibits negligible gate dependence. Remarkably, at *V*_g_ = −50V, *I*_SC_ depends linearly on incident power density P (Figure S6a). This indicates that *α* is close to 1 and near‐ideal photogenerated carrier separation efficiency is achieved due to the substantial conduction and valence band offsets in the p–n regime. Moreover, the output power reaches 11.6 nW and also shows a linear dependence on incident power density P (Figure S6b).

To better understand the effect of gate voltage on the built‐in potential, time‐resolved *V*_OC_ were further measured for cycled periodic illumination (Figures 3c and S7). Figure 3d summarizes *V*_OC_ values for various light intensities and gate voltages, revealing a clear distinction between the p–n and n–n^+^ regimes. In the p–n regime and *V*_g_ = –50 V, *V*_OC_ remains nearly constant (~2 V) regardless of light intensity, indicating the built‐in potential is dominated by the substantial conduction and valence band offsets. In this ideal p–n regime, photogenerated carriers experience negligible interfacial accumulation, with nearly all contributing to the photocurrent (*α* ≈ 1), indicative of an ideal charge separation efficiency. In sharp contrast, at *V*_g_ = –40 V, *V*_OC_ increases with light intensity, indicating some photogenerated carriers are accumulated at the interface to enhance the built‐in potential via the photovoltaic effect [66]. This transition from a static, offset‐dominated field to a dynamic, accumulation‐enhanced field underscores the high degree of electrostatic tunability in our device. This pronounced sensitivity of the interfacial field to the local carrier population not only allows for electrostatic control but also suggests a strong potential for modulation by other external stimuli, such as molecular physisorption. For *V*_g_ > –30 V, *V*_OC_ also increases with light intensity but its value is significantly smaller than that in p–n regime, which can be clearly see from Figure 3e,f. Collectively, these results demonstrate that the built‐in potential in the InSe/SnS_2_ vdW heterojunction devices are highly tunable via electrostatic gating and light illumination. This establishes a robust platform for engineering high‐performance, self‐powered optoelectronic devices through external field modulation.

**FIGURE 4:**
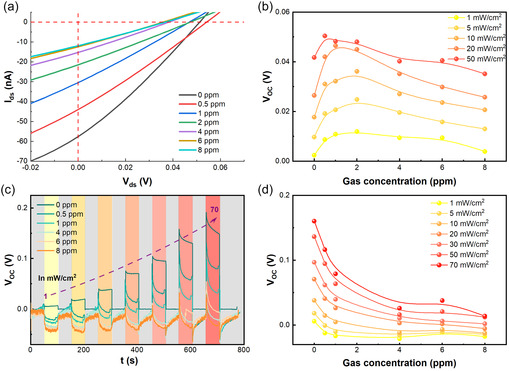
(a) *I*_ds_ *–V*_ds_ curves measured under a variety of NO_2_ gas concentrations at *V*_g_ = 0V and with a constant incident power density of 70 mW cm^−2^. (b) *V*_OC_ as a function of NO_2_ gas concentrations extracted from (a). (c) Time‐resolved *V*_OC_ under periodic illumination cycles measured under a variety of NO_2_ gas concentrations. (d) *V*_OC_ as a function of NO_2_ gas concentrations for a variety of indicant power densities extracted from (c).

To elucidate the influence of molecular adsorption on the self‐powered performance of the InSe/SnS_2_ vdW heterojunction devices, Figure 4a plots *I*_ds_ *–V*_ds_ curves measured under a variety of NO_2_ gas concentrations at *V*_g_ = 0V and with a constant incident power density of 70 mW cm^−2^. *I*_SC_ decreases with increasing NO_2_ gas concentration and its value decreases from 58 to 12 nA when the NO_2_ gas concentration is increased from 0 to 8 ppm (Figure S8). In contrast, *V*_OC_ shows a non‐monotonic trend. Its value initially increases with NO_2_ concentration, peaks at 0.5 ppm and then decreases when the NO_2_ concentration rises further. A similar trend was observed for each of the tested incident power densities with *V*_OC_ always peaking within the range of 0.5–2 ppm. However, the peak position is not fixed but shifts as a function of the incident power density (Figure 4b), highlighting the sensitivity of the junction to excitation conditions. This complex non‐monotonic behavior of *V*_OC_ suggests a competition between two opposing mechanisms. At *V*_g_ = 0V, the device operates in an n–n^+^ mode where the built‐in field is weak. The initial rise in *V*_OC_ at very low NO_2_ concentrations (<0.5 ppm) can be attributed to a surface passivation effect, where adsorbed molecules neutralize existing surface defects, thereby reducing Fermi level pinning and slightly increasing the effective built‐in potential. However, as the concentration increases, a competing suppressing effect begins to dominate, causing the subsequent decrease in *V*_OC_. The physical nature of this compete suppressing effect was further explored through dynamic measurements.

To explore this phenomenon in further details, Figure 4c plots time‐resolved *V*_OC_ under cyclic illumination measured for a variety of NO_2_ gas concentrations. The results provide clear evidence for the suppressing mechanism. Indeed, a clear trend of decreasing *V*_OC_ with increasing NO_2_ concentrations (Figure 4d) accompanied by pronounced transient responses upon illumination switching, indicative of dynamic carrier trapping and detrapping processes [43, 44, 49]. These slow response tails are indicative of dynamic carrier trapping and detrapping processes, confirming that the adsorbed NO_2_ molecules create localized electronic states that act as carrier traps. Indeed, density functional theory calculations indicate that localized defect states emerge within the bandgaps of both InSe [47] and SnS_2_ [48, 49] following NO_2_ adsorption. These adsorption‐induced defect states act as efficient recombination centers, capturing electrons or holes. This trapping mechanism also explains the dynamic equilibrium under continuous illumination. The energy from photons facilitates molecular desorption from the junction interface, which effectively empties these traps and restores mobile carrier density. The interplay between these two competing processes—carrier immobilization via molecular adsorption and carrier liberation through light‐induced desorption—establishes a dynamic equilibrium that dictates the strength and distribution of the internal electric field. This dynamic regulation, in turn, exerts a pivotal influence on charge separation dynamics and is directly manifested in the self‐powered photoresponse of the device.

At zero gate bias (*V*_g_ = 0 V), the vdW heterojunction operates in the n–n^+^ regime, distinguished by an exceptionally small conduction band offset (<0.03 eV). Within this operational regime, the *V*_OC_ exhibits minimal sensitivity to gate modulation and is predominantly determined by the density of photogenerated carriers (Figure 3e). The strong electron affinity of adsorbed NO_2_ molecules facilitates efficient electron trapping on the surfaces of InSe and SnS_2_, effectively reducing the reservoir of free photogenerated carriers available for conduction. As the concentration of NO_2_ increases, carrier immobilization intensifies, culminating in a marked suppression of both photocurrent and photovoltage. For example, as shown in Figure 4a, exposure to 8 ppm NO_2_ under an incident power density of 70 mW·cm^−2^ reduces the short‐circuit current (*I*_SC_) by nearly a factor of five and concomitantly lowers the *V*_OC_ three‐fold, from its peak value, 0.19 V to around 0.06 V.

**FIGURE 5:**
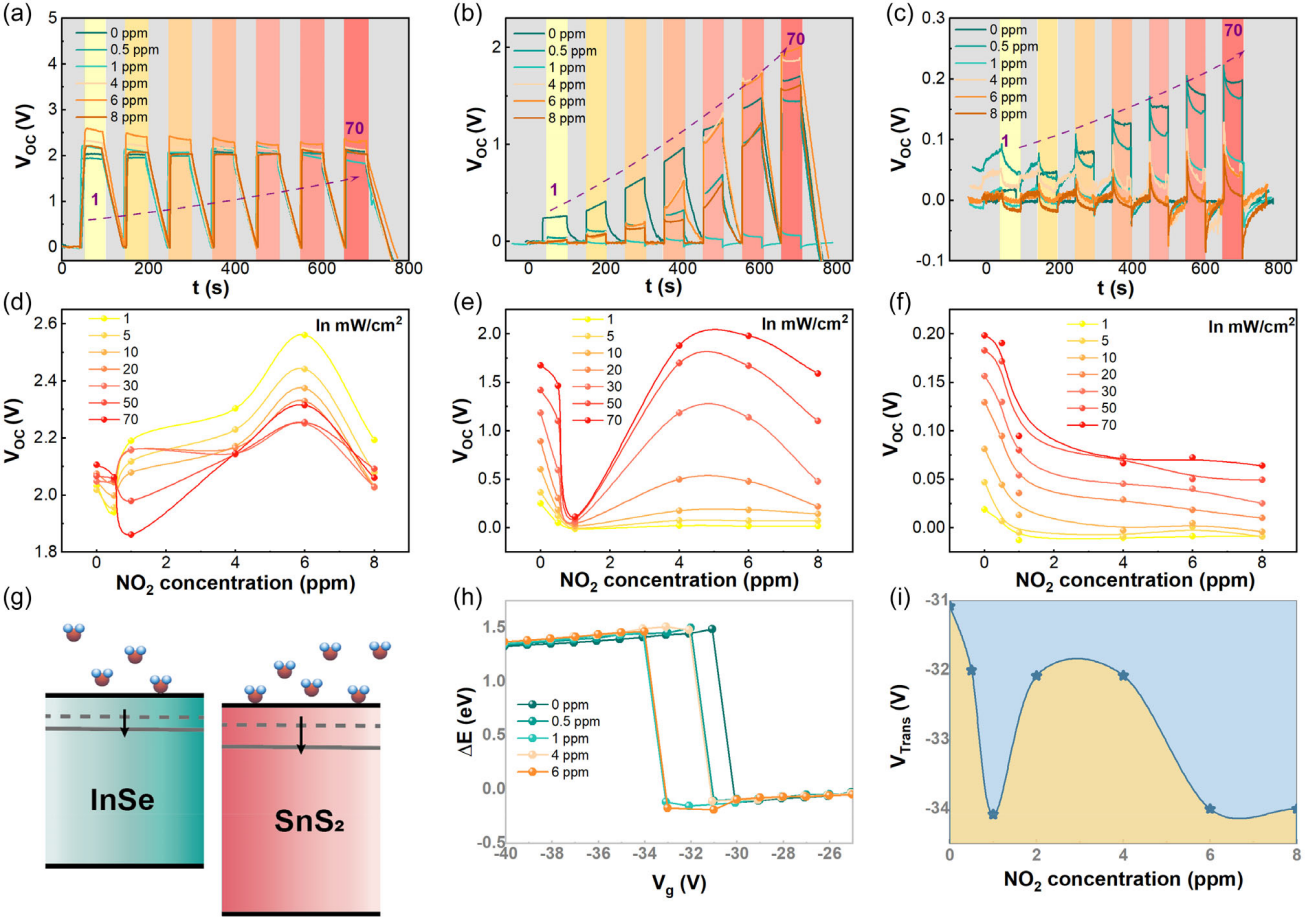
(a–c) Time‐resolved *V*_OC_ for periodic illumination cycles measured under a variety of NO_2_ gas concentrations at *V*_g_ = −50, −40, and −30 V respectively. (d–f) Summarized *V*_OC_ as a function of NO_2_ concentrations at *V*_g_ = −50, −40, and −30 V, respectively. (g) Schematic of the band diagram for NO_2_ molecules adsorbed on surface of InSe and SnS_2_. (h) The conduction band difference between InSe and SnS_2_ as a function of *V*_g_ when exposed to a variety of NO_2_ concentrations. (i) Summarized *V*_Trans_ as a function of NO_2_ concentrations.

In addition to carrier trapping, gas molecule adsorption on the surfaces of the vdW materials also induces charge transfer between the material surface and adsorbates, leading to Fermi level modification [46]. In vdW heterojunctions, the effect of molecular adsorption on *V*_OC_ or the built‐in potential becomes more complex due to interfacial band alignment between the constituent layers [67]. In fact, earlier reports show that NO_2_ adsorption enhances the *V*_OC_ of InSe/SnS_2_ vdW heterojunction devices [43]. However, the effect of NO_2_ adsorption was only investigated for specific gate voltage conditions, and it treated NO_2_ adsorption solely as a performance‐enhancing mechanism. Crucially, our experiments reveal the dual competitive nature of NO_2_—acting simultaneously as an electron acceptor and recombination center—and the dynamic evolution of this competition with applied gate voltage. Here, we will demonstrate how NO_2_ physisorption can precisely modulate the band alignment and built‐in potentials in self‐powered InSe/SnS_2_ heterojunction photodetectors, and provide a more comprehensive explanations and exploration of the system through electrostatic gating. To further investigate the interplay between the molecular adsorption and band alignment, Figure 5a–f shows the time‐resolved *V*_OC_ under periodic illumination cycles measured under a variety of NO_2_ gas concentrations at *V*_g_ = −50V, −40 V, and −30 V respectively. The results reveal three distinct operational regimes, whose behaviors can be consistently explained by a unified model based on the competition between two primary effects of NO_2_ physisorption: (1) efficiency enhancement via p‐i‐n structure formation, and (2) carrier loss via interfacial trapping and recombination.

### 2.1 Regime I (*V*_g_ = −50V): Enhancement‐Dominated Behavior

For *V*_g_ = −50V, the device operates in a strong p–n mode, as the ambipolar InSe layer effectively functions as a p‐type region while the SnS_2_ layer remains n‐type (Figure S10a). As shown in Figure 5a,d, *V*_OC_ is monotonically enhanced with NO_2_ physisorption, increasing with NO_2_ concentration and reaching a maximum value of 2.6V for a NO_2_ concentration of 6 ppm and an incident power density of 1 mW/cm^2^. In this regime, the initial built‐in field is so powerful that carrier separation is extremely efficient. The competing suppressing effect of carrier trapping is therefore largely suppressed. Moreover, upon exposure to NO_2_ gas, the adsorbed NO_2_ molecules extract electrons from both the InSe and SnS_2_ layers, leading to a reduction in electron concentration (collective electron withdrawal). The withdrawn electrons to become fixed negative charges (NO_2_^−^). These fixed charges create a strong, localized electric field that fully depletes the SnS_2_ layer at the interface, effectively widening the depletion region. Consequently, the structure evolves from a p–n junction to a p‐i‐n‐like configuration: the hole‐accumulated InSe acts as the p‐side, the NO_2_‐induced fully depleted SnS_2_ interface serves as the i‐region, and the unaffected SnS_2_ bulk remains the n‐side (Figure S10b). Thus, the carrier density in the original n‐type SnS_2_ will transform into a more intrinsic region due to carrier trapping by NO_2_ molecular. This improves charge separation efficiency, leading to the observed enhancement in the *V*_OC_.

### 2.2 Regime III (*V*_g_ ≤ −30 V): Trapping‐Dominated Behavior

In contrast, for *V*_g_ = −30V, the junction operates in a weak n–n^+^ mode. As seen in Figure 5c,f, *V*_OC_ monotonically decreases with NO_2_ physisorption. Here, the initial built‐in field is negligible. The adsorbed NO_2_ molecules primarily act as powerful recombination centers, capturing photogenerated carriers and drastically reducing their population. This carrier trapping and recombination effect is the dominant mechanism, leading to a consistent suppression of *V*_OC_.

### 2.3 Regime II (*V*_g_ = −40V): The Competitive Transition

Most interestingly, the intermediate *V*_g_ = −40V regime represents the critical transition between the two dominant effects. As shown in Figure 5b,e, *V*_OC_ exhibits a complex, nonmonotonic dependence on NO_2_ concentration. It initially decreases, reaching a minimum at ~1 ppm, and then rises to a peak at ~4 ppm before finally declining. This behavior vividly illustrates the competition: (1) At low concentrations (<1 ppm): The carrier trapping and recombination effect (supressing) is dominant, causing the initial drop in *V*_OC_. (2) At higher concentrations (1–4 ppm): The constructive effect of p‐i‐n structure formation begins to overpower the trapping loss, leading to the strong recovery and enhancement of *V*_OC_. (3) At very high concentrations (>4 ppm): Other secondary effects, such as increased scattering or trap saturation, may lead to the final decline.

It is also known that when a NO_2_ molecule is adsorbed onto the InSe and SnS_2_ surfaces, electrons transfer from InSe and SnS_2_ to NO_2_, causing the Fermi level of both materials to shift downward [46, 68, 69] (Figure 5g). To quantitatively investigate the effect of NO_2_ adsorption on the band alignment, Figure 5h shows the conduction band difference between InSe and SnS_2_ as a function of *V*_g_ when exposed to a variety of NO_2_ concentrations extracted from Figure S9. From Figure S9, one can clearly observe that NO_2_ adsorption significantly modifies SnS_2_ properties when *V*_g_ > −30 V, while substantial effects for InSe occur primarily at *V*_g_ < −30 V. This behavior leads to a nonmonotonic trend with increasing NO_2_ concentration. Interestingly, the band alignment of the vdW InSe/SnS_2_ heterojunction device can be controlled by NO_2_ concentrations and the *V*_Trans_ trends mirrors the *V*_OC_ behavior in regime II (Figure 5i), further confirm that the device's fundamental electronic structure is being dynamically modulated by the gas concentration. Therefore, the interplay between the gate voltage and NO_2_ physisorption defines the operational mode. We also performed similar tests under three alternative gas conditions: ambient atmosphere, 8.0 ppm H_2_S, and 160.0 ppm NH_3_ (Figure S11). The results clearly demonstrate that NO_2_ produces substantially more pronounced effects in comparison with the other gases. The reversibility, stability, and endurance of the NO_2_ physisorption‐induced modulation were also evaluated. Figure S12a shows the time‐resolved *V*_OC_ under periodic illumination cycles measured at *V*_g_ = 0 V in the presence of 4 ppm NO_2_. The device exhibits stable and repeatable photo‐responses, with *V*_OC_ consistently recovering to its initial baseline value after each cycle, indicating negligible baseline drift and good reversibility under illumination. In addition, Figure S12b presents the time‐resolved *I*_SC_ measured under repeated NO_2_ exposure/desorption cycles at a fixed illumination power density of 70 mW/cm^2^. The *I*_SC_ reproducibly returns to its original value after each gas removal step**,** demonstrating reliable recovery behavior and endurance over multiple gas cycles**.** These results confirm that the physisorption‐induced modulation is reversible and stable, which helps validate the erasable‐memory characteristics of the device. We also compared the self‐powered performance of our InSe/SnS_2_ vdW photodetector with other self‐powered vdW photodetectors mentioned in the introduction. Our analysis revealed that the *V*_OC_ values for SnS_2_/ReSe_2_, PdSe_2_/MoTe_2_, and InSe/WSe_2_/SnS_2_ self‐powered vdW photodetectors are approximately 0.2, 0.02, and 0.5 V, respectively [15, 16, 17]—significantly lower than the *V*_OC_ achieved by our InSe/SnS_2_ device (2.6 V). In addition, our vdW photodetector exhibits a nearly ideal carrier separation efficiency (α ≈ 1), surpassing those of the PdSe_2_/MoTe_2_ (0.92) [16] and InSe/WSe_2_/SnS_2_ (0.935) [17] photodetectors. Beyond these performance metrics, our device offers a distinct functional advantage. Conventional self‐powered vdW photodetectors rely on static built‐in electric fields arising from fixed band alignments, asymmetric contacts, or permanent chemical doping, resulting in predefined and non‐reconfigurable device behavior. In contrast, the InSe/SnS_2_ heterojunction presented here introduces a dynamically tunable self‐powered platform, in which the built‐in potential can be continuously and reversibly modulated through the combined action of electrostatic gating and NO_2_ molecular physisorption. This dual‐control strategy enables reversible tuning of the open‐circuit voltage, carrier separation efficiency, and operational polarity within a single device, a capability that is not demonstrated in previously reported self‐powered vdW photodetectors.

## 3 Conclusion

In summary, we demonstrate self‐powered vdW InSe/SnS_2_ photodetectors whose operational mode is defined by the interplay between gate voltage and NO_2_ physisorption. In the moderate p–n regime, NO_2_ adsorption selectively tailors the band alignment and built‐in potential, enabling unprecedented external control over device functionality. This approach establishes targeted molecular physisorption as a versatile strategy for engineering charge separation and transport in vdW heterostructures, paving the way for high‐performance, all‐in‐one devices based on self‐powered photodetectors.

## 4 Experimental Section/Methods

### 4.1 Device Fabrication and Characterization

In this study, SnS_2_, InSe, and h‐BN flakes were mechanically exfoliated onto polydimethylsiloxane (PDMS) substrates using a standard tape‐assisted method. First, h‐BN nanosheets were transferred onto a Si substrate with a 600 nm‐thick SiO_2_ layer via dry transfer. Subsequently, the exfoliated InSe and SnS_2_ flakes were carefully aligned and transferred onto the h‐BN surface using PDMS films. Au electrodes with a thickness of 35 nm were fabricated using standard ultraviolet lithography followed by thermal evaporation. Mechanically exfoliated vdW‐layered materials SnS_2_ and InSe were characterized via optical microscope (Olympus BX51), AFM (Bruker MM8), Raman spectra (Bruker Senterra with an excitation wavelength of 532 nm), and SKPM. Electrical measurements were performed using Keithley 2400 SourceMeters to apply biases and gate voltages while monitoring output currents. For photoresponse characterization, the devices were illuminated by lasers at varying power densities. The open‐circuit voltage (*V*_OC_) and short‐circuit current (*I*_SC_) were measured directly using a Keithley 2182 Nanovoltmeter and a Keithley 6216 nm, respectively.

## Supporting Information

Additional supporting information can be found online in the Supporting Information section. **Supporting Figure S1**: Topographical AFM images and corresponding height profiles of InSe and SnS_2_. **Supporting Figure S2** **:** (a‐c) Gaussian fitting of SKPM characterizations to extract of the work functions of individual InSe, SnS_2_, and their overlapping regions respectively. **Supporting Figure S3** **:** (a, b) I_ds_–V_g_ curves of the individual InSe and SnS_2_ layer to extract their Fermi levels before contact respectively. **Supporting Figure S4** **:** Summarized I_SC_ as a function of incident power density extracted from Figure 2d. **Supporting Figure S5** **:** (a) Magnified I_ds_–V_ds_ curves of a InSe/SnS_2_ vdW heterojunction device measured at a variety of V_g_ under 365 nm light illumination with an incident power density of 70 mW·cm^−2^. (b) Summarized V_OC_ extracted from (a). **Supporting Figure S6** **:** (a, b) I_SC_ and output power as a function of incident powder density at V_g_ = ‐50V, respectively. **Supporting Figure S7** **:** Time‐resolved V_OC_ measured with different incident power densities at V_g_ = ‐50 V and ‐40V. **Supporting Figure S8** **:** I_SC_ as a function of NO_2_ gas concentration extracted from Figure 4a. **Supporting Figure S9** **:** (a, b) I_ds_‐V_g_ curves of the individual InSe and SnS_2_ layer when exposed to a variety of NO_2_ concentrations to extract their Fermi levels before contact respectively. **Supporting Figure S10** **:** (a) schematic illustrating the band bending before and (b) after NO_2_ adsorption in Regime I (V_g_ = ‐50V). **Supporting Figure** **S** **11** **:** Time‐resolved V_OC_ under periodic illumination cycles measured under ambient condition, 8 ppm NO_2_, 8 ppm H_2_S, and 160 ppm NH_3_ at V_g_ = 0V. **Supporting Figure S12** **:** (a) Time‐resolved V_OC_ under periodic illumination cycles measured under 4 ppm NO_2_, where the incident power density is 30 mW/cm^2^. (b) Time‐resolved I_SC_ under periodic gas cycles measured under 70 mW/cm^2^ light illumination.

## Author Contributions

**Ze Cao:** data curation (lead), investigation (lead). **Mohamed Abid:** formal analysis (equal), methodology (equal). **Cormac Ó Coileáin:** formal analysis (equal), writing – review & editing (equal). **Fengjiang An:** conceptualization (equal), investigation (equal), supervision (equal). **Ching‐Ray Chang:** formal analysis (equal), writing – review & editing (equal). **Yuh‐Renn Wu:** formal analysis (equal), writing – review & editing (equal). **Han‐Chun Wu:** conceptualization (lead), data curation (lead), formal analysis (lead), Funding aquisition (lead), investigation (lead), project administration (lead), resources (lead), supervision (lead), validation (lead), writing – original draft (lead), writing – review & editing (lead).

## Funding

This work was supported by the National Natural Science Foundation of China (62374017, 61874010), the Science and Technology Innovation Program for Creative Talents in Beijing Institute of Technology (2017CX01006), the National Science and Technology Council, Taiwan (R.O.C.), under grant No. 112‐2221‐E‐002‐214‐MY3 and 112‐2221‐E002‐215‐MY3, and the Leap fellowship of the Foundation for the Advancement of Outstanding Scholarship.

## Conflicts of Interest

The authors declare no conflicts of interest.

## Supporting information

Supplementary Material

## Data Availability

The data that support the findings of this study are available from the corresponding author upon request.
