# Supplementary material for: Dynamic Control of Band Alignment and Built‐In Potential in High Performance Self‐Powered InSe/SnS2 Van der Waals Photodetectors via Gas Molecular Physisorption
Source: Small Sci. 2026 Mar 6;6(3):e202500616. doi: 10.1002/smsc.202500616 (PMC12970161; doi:10.1002/smsc.202500616)
Supplement: Supplementary file 1 — Supplementary Material [file SMSC-6-e202500616-s001.pdf]

Supporting Information

Dynamic control of band alignment and built-in potential in high performance self-powered $InSe/SnS_2$ van der Waals photodetectors via gas molecular physisorption

Ze Cao, Mohamed Abid, Cormac Ó Coileáin, Fengjiang An*, Ching-Ray Chang, Yuh-Renn Wu, and Han-Chun Wu*

Band offset calculation:

To calculate the band offset, we need to calculate the band density of states N_C, mobility μ, carrier density (n_e) for InSe and SnS_2 respectively.

(1) Calculating conduction band density of states N_C:

$N_C = \frac{2(2\pi m_e KT)^{3/2}}{h^3}$ Here, m_e represents the effective electron mass of a two-dimensional semiconductor material, and h is the Planck constant, with a value approximately equal to 6.62607015×10^{-34}J·s.

(2) Calculating mobility μ:

$\mu = \frac{L}{WC_{ox}V_{ds}} \times \frac{dI_{ds}}{dV_G}$ Here, C_{ox} represents the capacitance size of the device substrate, $V_{ds}$is the bias voltage used when testing the transfer characteristics of the material, and $\frac{dI_{ds}}{dV_G}$ is the slope of the material transfer characteristic curve.

(3) Calculating carrier density n_e:

$n_e = \frac{L}{RAe\mu}$ Here, Lrepresents the length of the material, Rrepresents the material's resistance, $A = W \times d$ represents the cross-sectional area of the material（where W and d are the width and thickness of the material respectively）, and μ represents the material's mobility.

(4) Calculating band offset:

$n_e = N_C e^{\frac{-(E_C-E_F)}{KT}}$. Here, K represents the Boltzmann constant, with a value approximately equal to 1.380649×10^{-23} J/K. T denotes the Kelvin temperature, which is approximately 300K in the testing environment of this work.

In this work, we measured the transfer characteristic curves (I_{ds}-V_g) of InSe and SnS_2 materials with a

bias voltage of 4V. By taking the first derivative of the curves, we obtained the mobilities of the two materials. Using the above formulas, we in turn derived the values of $E_C - E_F$ and $E_V - E_F$ for the two materials (it should be noted that due to the bipolar nature of InSe, different V_g regions require separate calculations according to the corresponding polarity formulas). Finally, we obtained the relationship curves of ΔE_C and ΔE_V with the gate voltage, which is shown in Figure 3a of the manuscript.

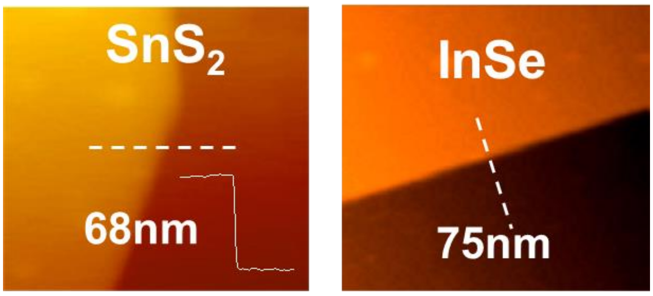

Figure S1. Topographical AFM images and corresponding height profiles of InSe and SnS_2.

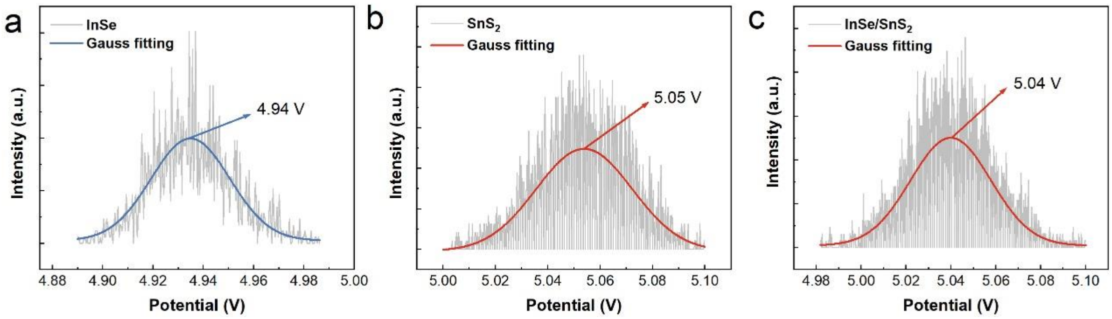

Figure S2. (a-c) Gaussian fitting of SKPM characterizations to extract of the work functions of individual InSe, SnS_2, and their overlapping regions respectively.

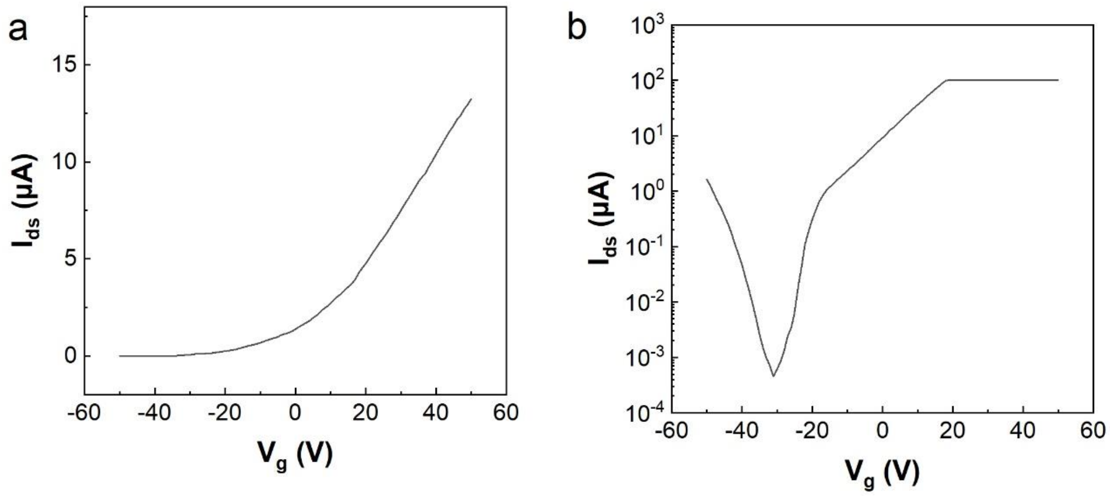

Figure S3. (a, b) I_{ds}-V_g curves of the individual InSe and SnS_2 layer to extract their Fermi levels before contact respectively.

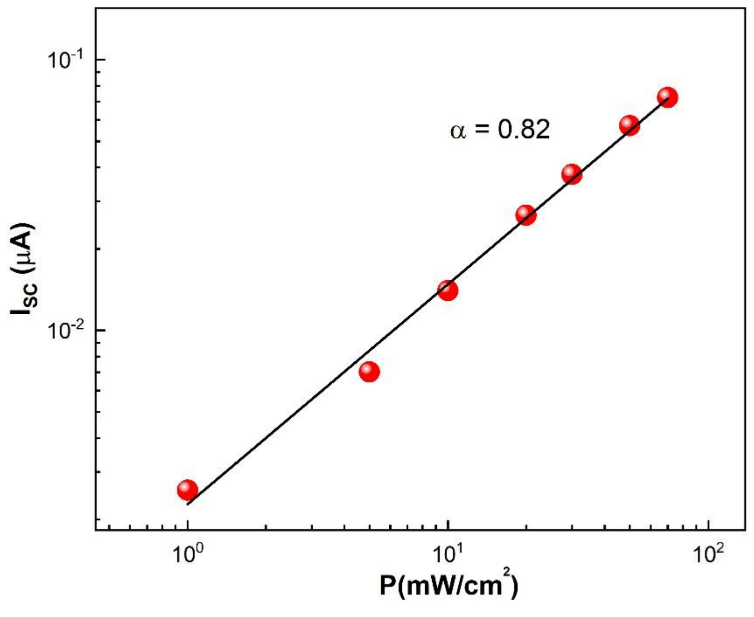

Figure S4. Summarized I_{SC} as a function of incident power density extracted from Figure 2d.

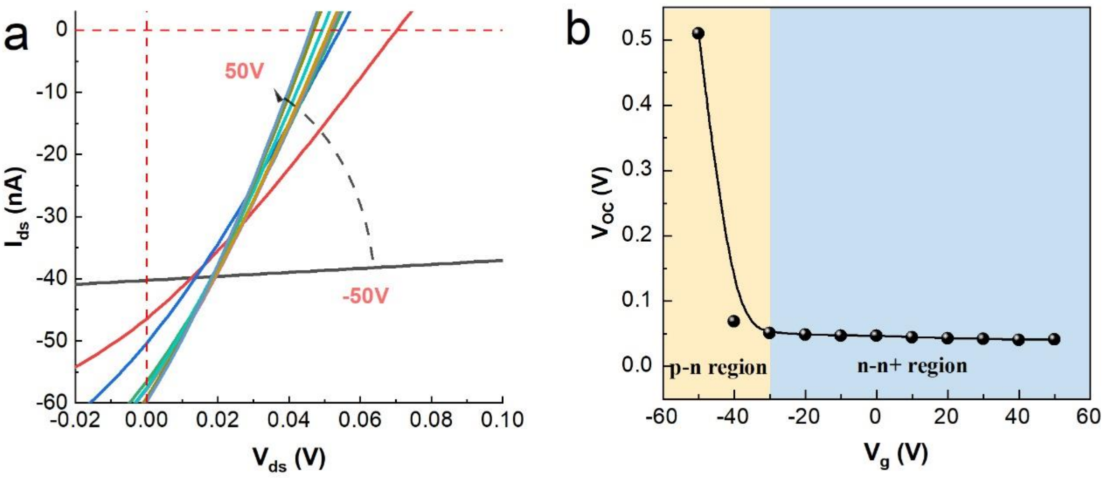

Figure S5. (a) Magnified I_{ds}-V_{ds} curves of a InSe/SnS_2 vdW heterojunction device measured at a variety of V_g under 365 nm light illumination with an incident power density of 70 mW·cm^{-2}. (b) Summarized V_{OC} extracted from (a).

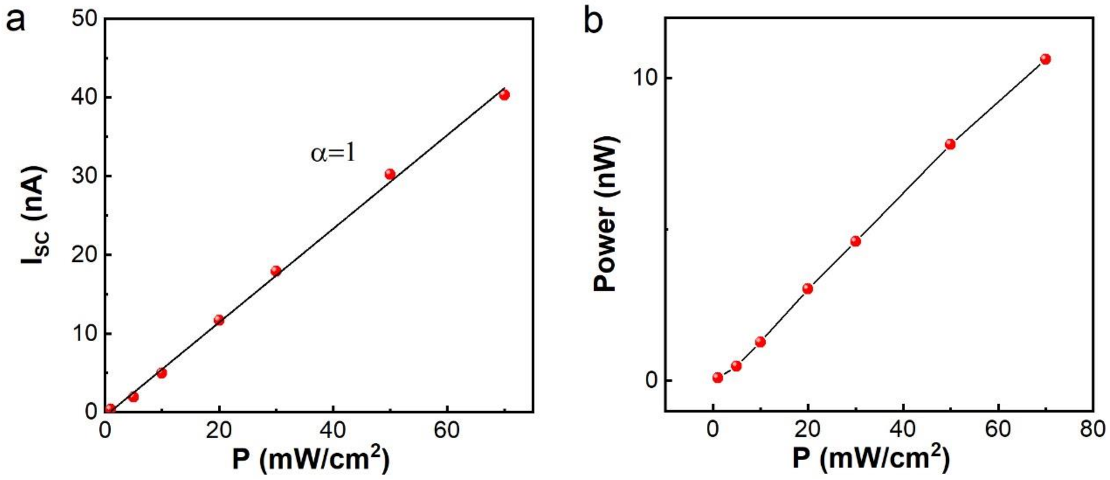

Figure S6. (a, b) I_{SC} and output power as a function of incident powder density at V_g =-50V respectively.

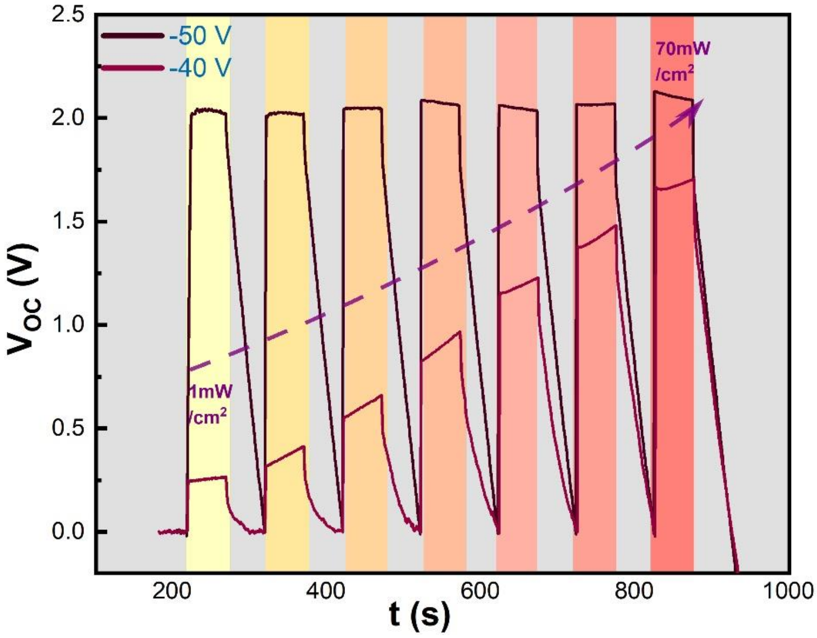

Figure S7. Time-resolved V_{OC} measured with different incident power densities at V_g = -50 V and -40V.

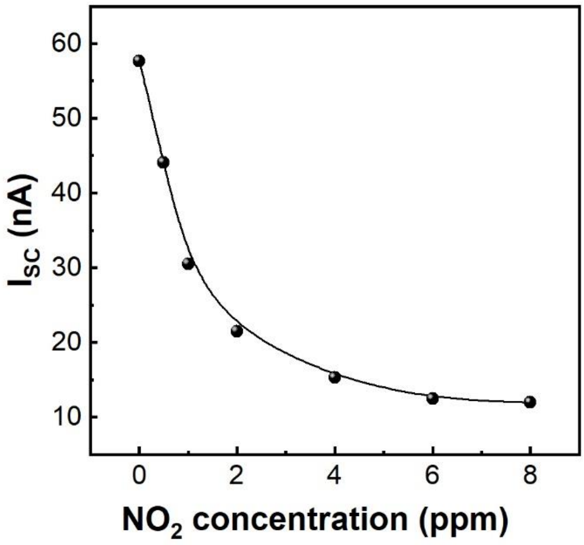

Figure S8. I_{SC} as a function of NO_2 gas concentration extracted from Figure 4a.

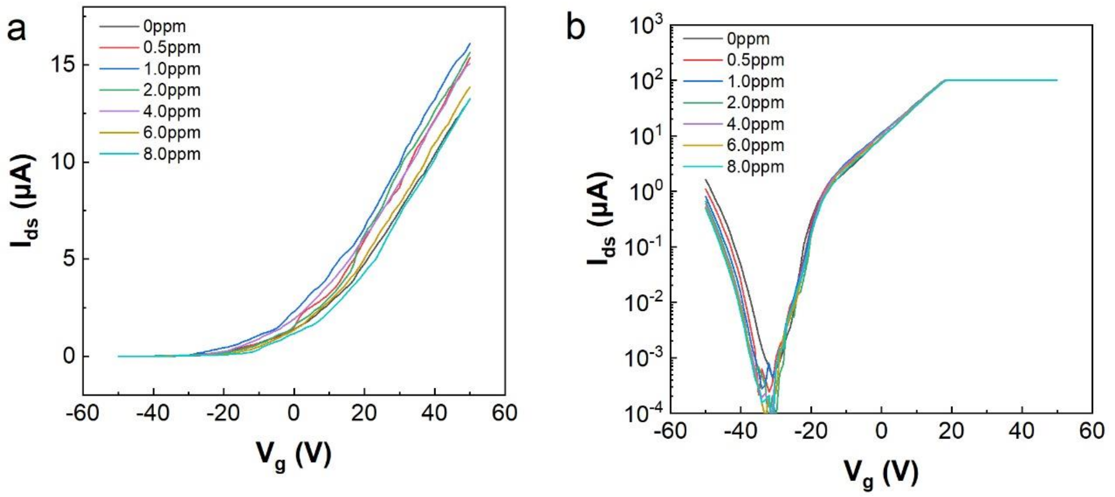

Figure S9. (a, b) I_{ds}-V_g curves of the individual InSe and SnS_2 layer when exposed to a variety of NO_2 concentrations to extract their Fermi levels before contact respectively.

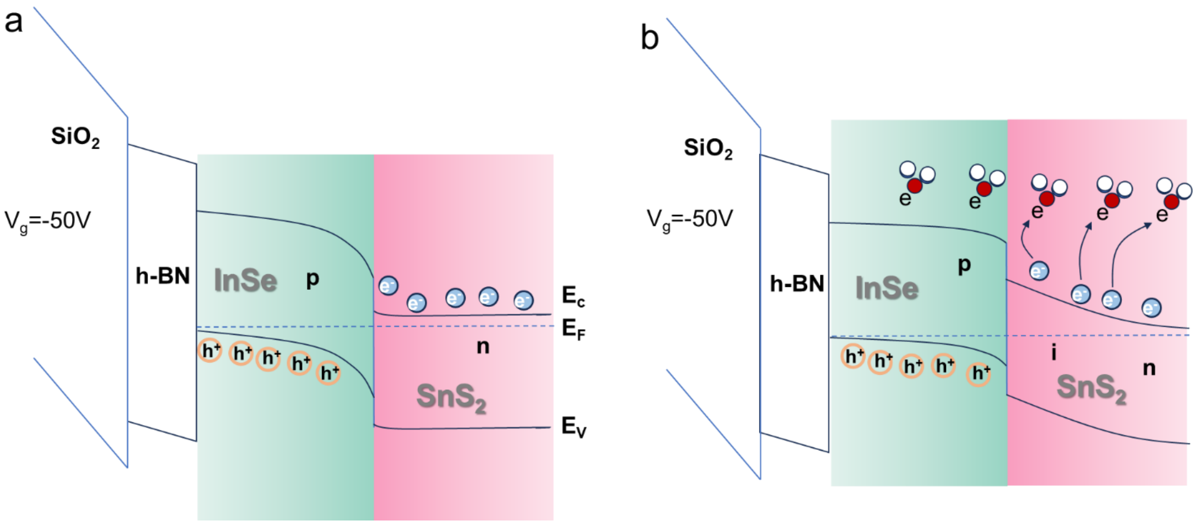

Figure S10. (a) schematic illustrating the band bending before and (b) after NO_2 adsorption in Regime I (V_g=-50V).

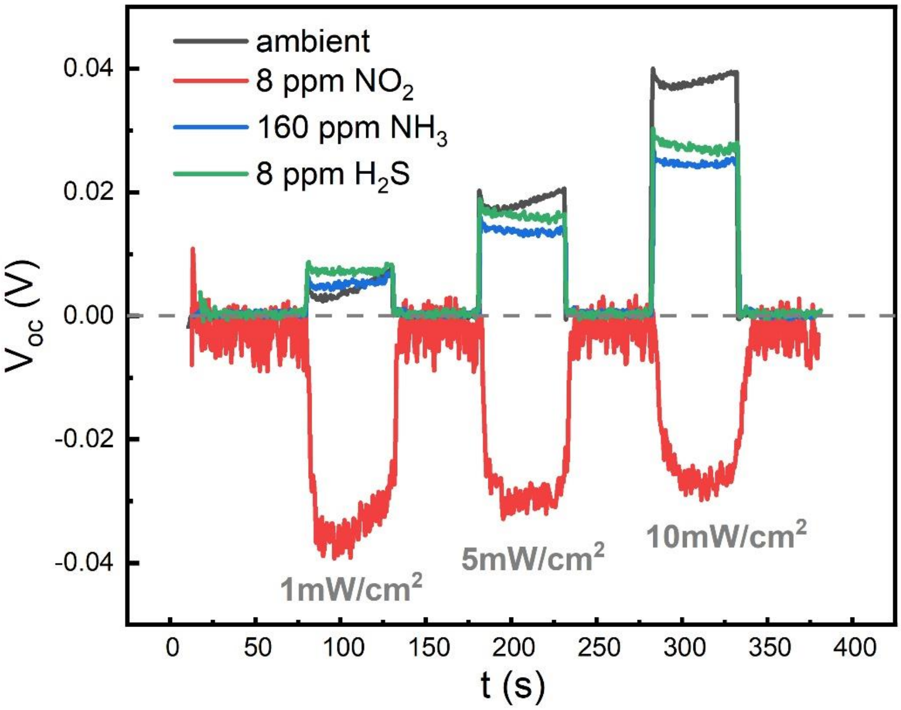

Figure 11. Time-resolved V_{OC} under periodic illumination cycles measured under ambient condition, 8 ppm NO_2, 8 ppm H_2S, and 160 ppm NH_3 at V_g=0V.

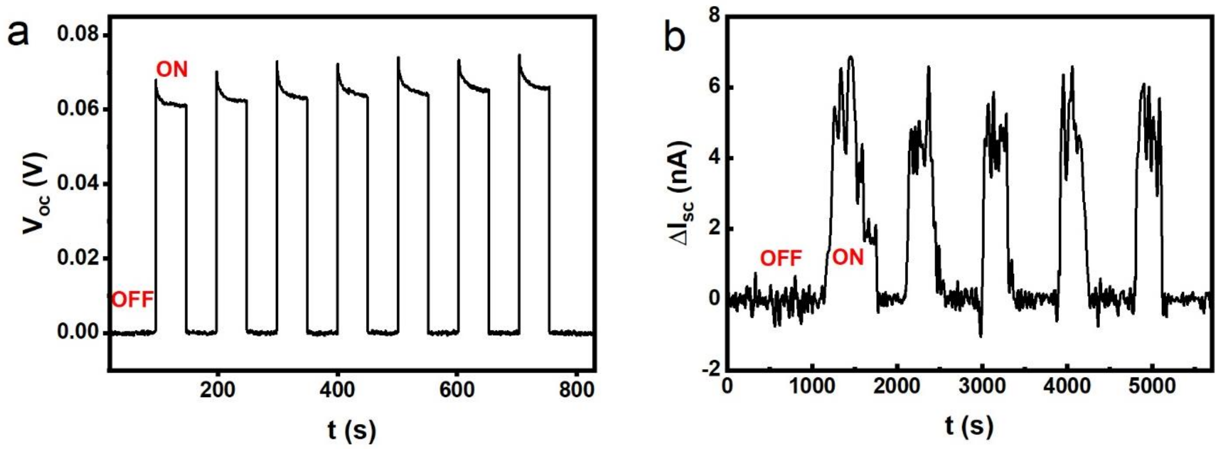

Figure S12. (a) Time-resolved V_{OC} under periodic illumination cycles measured under 4 ppm NO_2, where the incident power density is 30 mW/cm^2. (b) Time-resolved I_{SC} under periodic gas cycles measured under 70 mW/cm^2 light illumination.
